# A Single-Entity Method for Actively Controlled Nucleation and High-Quality Protein Crystal Synthesis

**DOI:** 10.1021/acs.analchem.3c00175

**Published:** 2023-05-27

**Authors:** Ruoyu Yang, Maksim Kvetny, Warren Brown, Edwin N. Ogbonna, Gangli Wang

**Affiliations:** Department of Chemistry, Georgia State University, Atlanta, Georgia 30302, United States

## Abstract

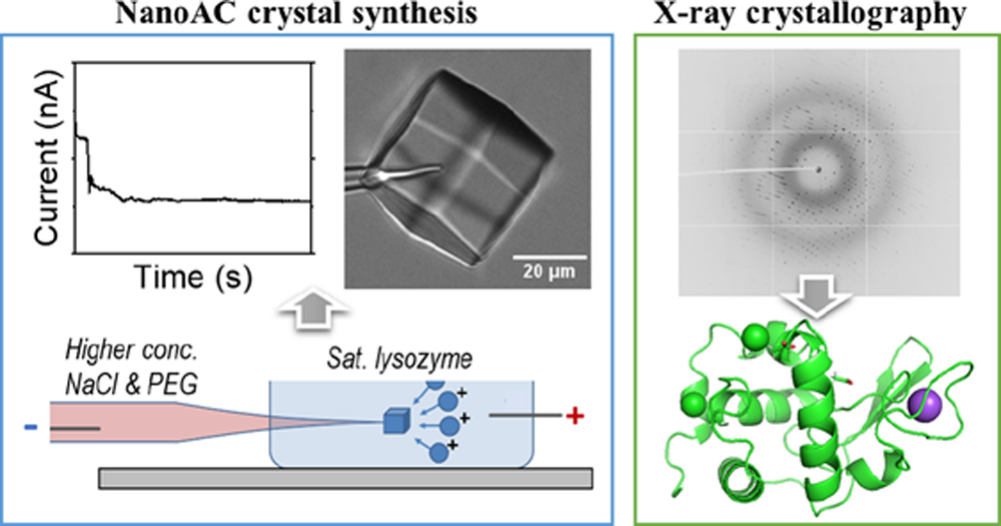

Lack of controls and understanding in nucleation, which proceeds crystal growth and other phase transitions, has been a bottleneck challenge in chemistry, materials, biology, and other fields. The exemplary needs for better methods for biomacromolecule crystallization include (1) synthesizing crystals for high-resolution structure determinations in fundamental research and (2) tuning the crystal habit and thus the corresponding properties in materials and pharmaceutical applications. Herein, a deterministic method is established capable of sustaining the nucleation and growth of a single crystal using the protein lysozyme as a prototype. The supersaturation is localized at the interface between a sample and a precipitant solution, spatially confined by the tip of a single nanopipette. The exchange of matter between the two solutions determines the supersaturation, which is controlled by electrokinetic ion transport driven by an external potential waveform. Nucleation and subsequent crystal growth disrupt the ionic current limited by the nanotip and are detected. The nucleation and growth of individual single crystals are measured in real time. Electroanalytical and optical signatures are elucidated as feedbacks with which active controls in crystal quality and method consistency are achieved: five out of five crystals diffract at a true atomic resolution of up to 1.2 Å. As controls, those synthesized under less optimized conditions diffract poorly. The crystal habits during the growth process are tuned successfully by adjusting the flux. The universal mechanism of nano-transport kinetics, together with the correlations of the diffraction quality and crystal habit with the crystallization control parameters, lay the foundation for the generalization to other materials systems.

## Introduction

The majority of high-resolution structures of biomacromolecules are determined by single-crystal X-ray crystallography. Crystals with high diffraction quality are prerequisites and often the bottleneck.^1,2^ Neutron scattering offers the unique advantage of resolving proton location but requires much larger high-quality crystals due to the lower signal intensity.^3^ Electron microscopy (EM) techniques have advanced tremendously in recent years. However, atomic resolutions (about 1.5–2.0 Å or better) remain rare, which leads to ambiguities in the interpretations of the corresponding properties and functions.^4,5^ Single-particle EM also faces fundamental limits in analyzing smaller unit cells as well as practical obstacles such as the requirements in specialized data treatment and instrument availability. For X-ray and neutron crystallography, the uncertainties and wastes are overwhelming because crystals diffracted up to atomic resolution remain extremely difficult to synthesize despite the significant investments in human time and resources on the screening of the chemical space. Crystallization methods with predictable outcomes remain to be developed.

The asynchronous and dynamic nature of nucleation is a fundamental challenge for the classic biomacromolecule crystallization methods, which are ensemble-based.^6−8^ Nucleation is an energy uphill process involving the assembly of individual molecule of interest into nuclei up to a critical size. Further growth after nucleation is thermodynamically favorable. The molecular assemblies such as pre-nucleation clusters and dense liquid domains during nucleation have attracted significant research interests, but their dynamics remains enigmatic and widely recognized to govern the fate of the subsequent crystal growth.^9−16^ Overall, the crystallization process involves the transition of the chemical system in a classic phase diagram, importantly identifying a metastable zone between the unsaturated stable zone and precipitation zone. The thermodynamics of the system can be described by the chemical potential in eq 1:
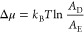
1where *k*_B_ is the Boltzmann constant, *T* is the temperature, and *A*_D_ and *A*_E_ are the activity of the analyte molecule (concentration is often used in approximation by omitting the correction by activity coefficient) and the activity at equilibrium (i.e., the solubility of the target analyte), respectively. During the whole crystallization process, both *A*_D_ and *A*_E_ will vary in time and space due to the mass exchange between the sample and the precipitant solutions. For example, nucleation at location 1 at time 1 will change the *A*_D_ in the surrounding, which affects subsequent growth or other nucleation events. The *A*_E_ can also change continuously because of the exchange of solvent and/or precipitants. Multiple nucleation events with different start times will obviously result in heterogeneous crystal products in ensemble systems. Because the growth is sustained by the concentration gradients localized around individual nuclei, the quality of individual crystals cannot be controlled in ensemble systems. Generally speaking, higher supersaturation in a precipitation zone is necessary for spontaneous nucleation. However, subsequent crystal growth prefers lower supersaturation in a narrowly defined metastable zone. The ability to suppress excessive nucleation is especially critical to growing larger high-quality crystals for stronger diffraction signals. It is important to emphasize that the changes in supersaturation induced by mass transport, occurring in a sample solution, are mechanistically different from the heterogeneous nucleation on the electrode surface driven by the faradic process in electrocatalysis, electrosynthesis, collisional reactions, or other electrochemical systems.^17−20^

Trial-and-error approaches are adopted universally to strike suitable thermodynamic and kinetic conditions, for example finding a combination of parameters for the chemical system to stay within the metastable zone after the dynamic nucleation process. High-throughput screening expedites the process by varying multiple parameters in parallel. Within each individual trial, however, diffusion driven by concentration gradient is still the main mechanism for mass exchange, which means the kinetics cannot be controlled in situ, and the start point depends on the stochastic individual nucleation events. Noteworthily, the same obstacles hinder the controls in crystal habit and morphology and thus the corresponding properties that are important for drug development, materials synthesis, and manufacturing in the pharmaceutical and material industry.^21−23^

**Scheme 1:**
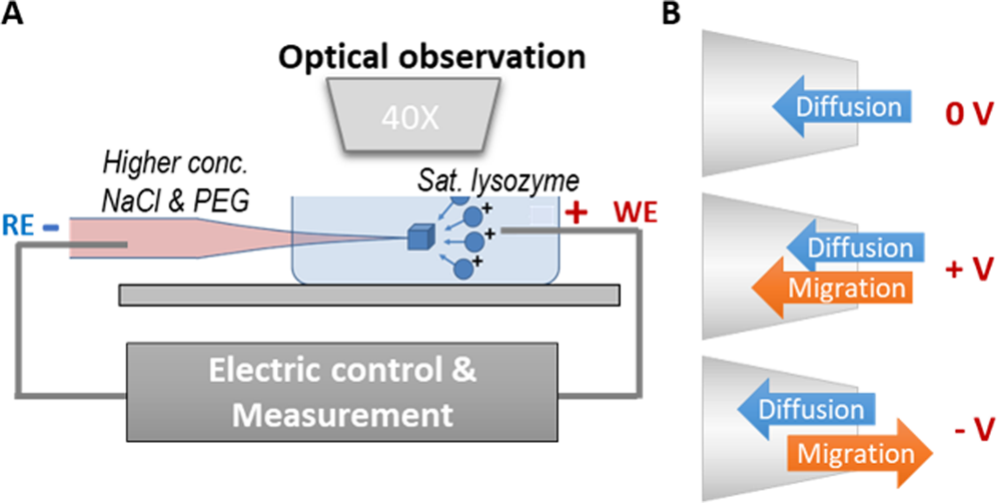
(A) Experimental Setup of Single Entity Crystallization with a Single Nanopipette. (B) Diffusion and Migration Directions of Lysozyme Molecules (Positive Charged) under Different Potential Bias. WE and RE Refer to Working and Reference Electrodes, Respectively

Single asymmetric solid-state nanopores and nanopipettes have attracted widespread interest in basic research as well as stochastic single entity analysis, nucleic acid sequencing, and other Coulter counter-type sensing applications.^24−29^ The spatially confined and temporally resolved transport properties offer intrinsic advantages and unprecedented capabilities to control individual nucleation and crystal growth processes because localized reagent delivery is limited by the most restrictive nanotip region and can be measured from the ion transport current.^30−33^ Our group has undertaken a combined experimental and simulation approach to quantitate the hysteresis charges,^34−36^ local electrical field effects of surface charges,^37−39^ and electroosmotic flow,^40,41^ among other time-dependent features over the well-known steady-state ion current rectification (ICR) property.^42−44^ Those fundamental insights lay the foundation for the single entity method reported herein, named NanoAC highlighting *Nanoscale Active Controls.* Hen egg white lysozyme (HEWL) is used as a prototype (Scheme 1), with the hardware modified from our earlier report about insulin crystallization driven by pH gradient.^45^ Critical advances are achieved to establish this new method in (1) true atomic resolution structures determined; (2) the semi-quantitative correlation between the kinetics of nucleation and growth with the diffraction quality; (3) the capability to control not just the crystal size but to tune the crystal habit; and (4) the successes with a universal and generalizable precipitant system: the gradients of electrolyte ions and polyethylene glycol (PEG) that are widely adopted for various biomacromolecule crystallizations.

## Methods

### Crystallization

All solutions were prepared in a 0.1 M acetate buffer pH 4.8, consisting of acetic acid (HAc) and sodium acetate (NaAc). HEWL (≥ 90%, Sigma-Aldrich) was dissolved in the acetate buffer at a concentration of 50 mg/mL as the stock solution. The precipitant solution contains 10% wt of α,ω-dicarboxylic polyethylene glycol (COOH-PEG-COOH, M.W.: 3.5 kDa) and 2 M sodium chloride (NaCl) in the acetate buffer. Immediately prior to crystallization experiments, equal volumes of the stock solution and a 1.1 M NaCl acetate buffer solution were mixed and centrifuged at 15,000 rpm for 10 min. The supernatant solution is used as the sample solution, which remains stable without spontaneous nucleation or crystal growth throughout the duration of nanopipette experiments (up to 1 week at room temperature). Typically, a 20 μL droplet of the sample solution is deposited in an in-house made cell under the microscope light path. The nanopipette is backloaded with the precipitating solution and inserted into the sample droplet. The sample is sealed by placing a coverslip on top of the chamber to ensure insignificant volume changes during the nanopipette experiments. The working electrode is a silver/silver chloride (Ag/AgCl) wire inserted in the sample droplet and another Ag/AgCl wire inside the capillary as a reference counter electrode.

### Instrumentation and Data Analysis

Nanopipettes in two size ranges, about 40 and 150 nm radius determined by conductivity measurements, are used (Table S1). The imaging and electroanalytical setup and the preparation and characterization of nanopipettes have been described previously.^34,37−41,45^ Further details are provided in the Supporting Information. Briefly, electroanalytical current and potential were recorded with a Dagan Chem-Clamp Amplifier (including a pre-amp head stage 100 M) controlled by the LabVIEW program. The potential was adjusted manually using the amplifier while identifying suitable conditions. Electroanalytical data were processed using Origin. All optical images were taken using an Olympus BX51WI equipped with a Lumenera INFINITY 3S monochrome camera and an Olympus LUMPlanFLN 40× objective (NA 0.80). Optical images were processed with ImageJ 1.48 including Micro-Manager 1.4.22.

X-ray diffraction data were collected at 100 K from beamline 8.2.1 (λ = 1.0000 Å) at the Advanced Light Source (ALS, Berkeley, California). Diffraction data were processed with the HKL2000 program package.^46^ The CCP4i2 suite was used to treat the Scalepack files.^47^ Molecular replacement and refinement were performed using Phenix 1.20^48^ and Coot 0.9.2.^49^ The electron density map and protein structure were prepared with Coot and PyMOL.

## Results and Discussion

### Three Distinct Stages of Phase Transitions

**Figure 1:**
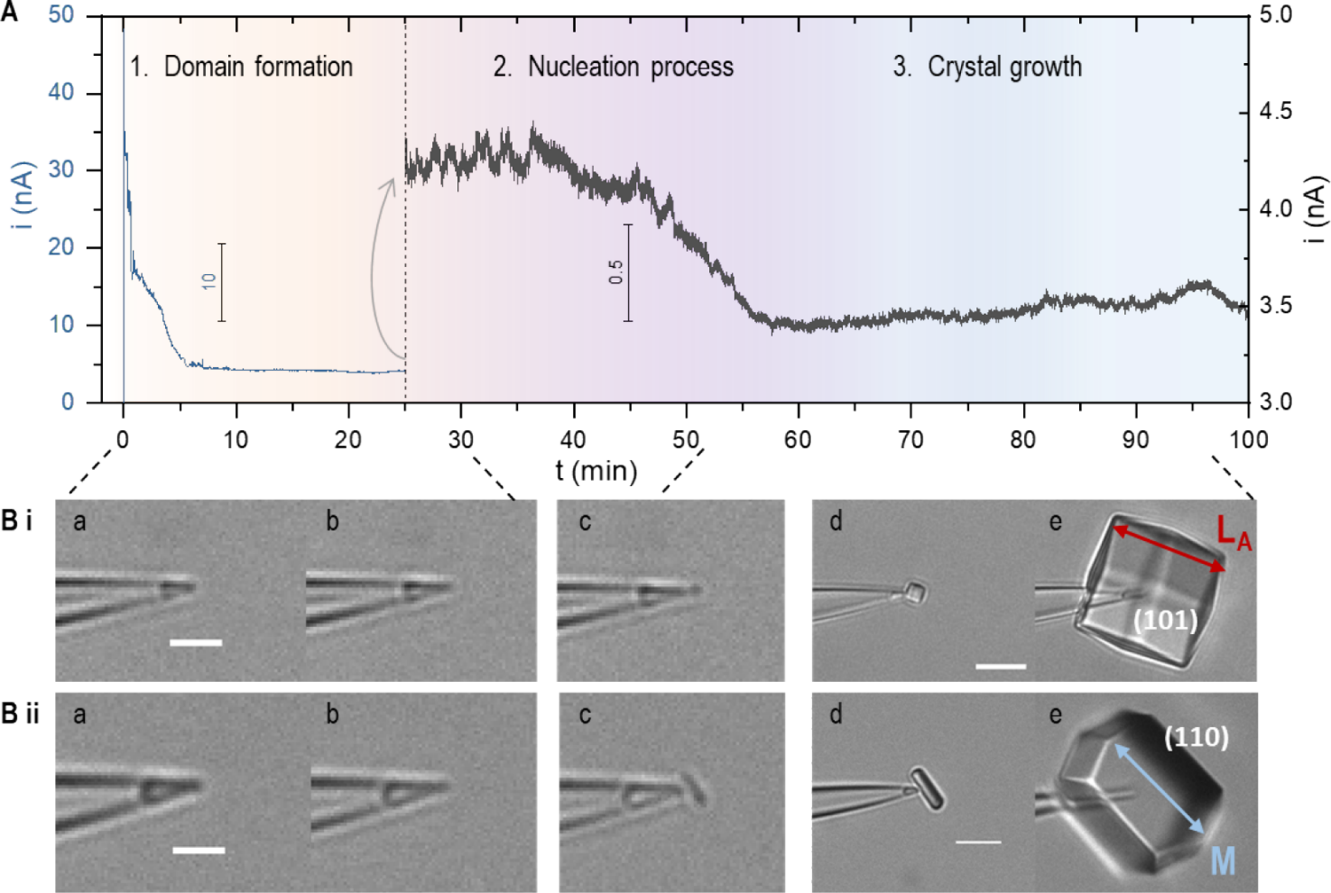
Phase transitions during lysozyme crystallization. (A) Electric current features of three distinct phase transition periods. The current–time traces were measured with a 40 nm-radius pipette. The potential bias (reference/ground electrode inside the nanopipette, RE) was held −0.1 V to suppress uncontrolled mixing during nanopipette insertion. The potential was switched to +1 V at time zero and held constant afterward. (B) Two time-lapse brightfield image series (i and ii) with lattice aligned in different orientations using 150 nm-radius pipettes. The scale bars are 4 μm in a–c and 10 μm in d and e.

Three stages of phase transitions are resolved during the single nanopipette HEWL crystallization: liquid domain formation, nucleation, and crystal growth. Representative electroanalytical and optical features are presented in Figure 1. The experimental setup is sketched in Scheme 1A, and three scenarios in Scheme 1B illustrate the relative contributions from migration and diffusion. HEWL has a pI of 11.36 and is positively charged at pH 4.8.^50^ A negative bias, typically −0.1 V, is first applied when the two solutions make contact upon the nanopipette insertion. In this pre-conditioning period, migration counters the diffusion to suppress uncontrolled phase transition since diffusion happens during the hardware setup. An example is in Figure S1, where phase transition is only induced up to about 2 min until sufficient time and positive potential are applied. The voltage-controlled pre-condition ensures highly consistent initial states, which are of paramount importance to determine the kinetics, especially at the early stage. Throughout this report, time zero is defined at the point when a positive voltage is applied.

The lysozyme concentration increases and its solubility decreases at the nanotip region over time due to the transport of HEWL and precipitants, inducing an increase in supersaturation, . *C*_tip_ and *C*_eq_ are the concentrations at the nanotip and at the equilibrium (saturation), respectively.^51^ Note both vary in time and space, different from the bulk ones in the sample droplet. An optically resolved domain forms and grows inside the nanotip (Figure 1B, a and b). Correspondingly, the electric current drops sharply, 10s nA within about 1 min, followed by a more gradual decrease (Figure 1A). The decrease in the baseline ionic current indicates a partial physical blockage or the decrease in apparent diffusion coefficients of the transported ionic species. This is the exact principle for resistive pulse sensing, for example, gene sequencing and Coulter counter-type sensors using protein ion channels or solid-state nanopores.^52,53^ The drastically lower yet “stable” current indicates that the new domain is ion-permeable but viscous or gel-like.^45^ The notion of the liquid domain is used in reference to the literature of the dense liquid domains, which are proposed as the first step in the two-step protein nucleation mechanism.^54,55^ Unlike the higher (bulk) supersaturations needed and adopted in macroscopic ensemble systems, the supersaturation is localized solely at the nanotip in the NanoAC system.

The liquid domain grows over time and ultimately extrudes outside the nanotip where nucleation completes (Figure 1B, a–c). Elongation of the extruded liquid domain or the formation of a lattice can be resolved optically after the edges reach sufficient sizes (more than 2 pixels or 0.5 μm). The emergence of crystalline structures and the evolution of crystal morphology are clearly demonstrated by the continuous monitoring of two crystals (Figure 1B, d and e) in which the lattice growth direction aligns differently from the view angle. Note that the images at earlier time points are limited by optical diffraction displaying individual pixels, and not indicating poor experimental quality. Since individual nanopipettes are inevitably heterogeneous with variations in nanogeometry and surface charge distribution, additional results from several smaller and larger nanopipettes (40 and 150 nm range based on conductivity characterization) are provided in Figures S3–S6. Given the main features are similar to those in Figure 1, those examples collectively cover other less common features, which may also result from the stochastic nature of individual nucleation events discussed later.

The efficacy of the NanoAC method is confirmed by the following control experiments. First, the sample solution itself does not nucleate or form crystals over the course of nanopipette experiments, normally 1 or 2 days. Second, the liquid domain and the following phase transitions would not occur or grow at a lower applied potential (Figure S1). In fact, the gel-like liquid domain will redissolve and disappear by adjusting the bias to less positive. On the other hand, without the precipitants or protein sample (Figure S2A–C), the liquid domain (and the following phase transitions) is not observed and the baseline current remains unchanged under otherwise comparable conditions (potential range, time). To induce phase transitions within a reasonable time, it is therefore favorable to prepare the sample to be close to saturation, so that supersaturation can be easily induced at the nanotip. Furthermore, from the current–time traces under different potentials in Figures S1 and S2, the migration flux driven by the applied electric field is readily differentiable from diffusional flux by comparing the measured current at different applied potentials. The streaming current at 0 V measures the diffusion under the concentration gradients across the nanotip. Technically, the current would not change the bulk concentrations or solution pH based on simple calculations: a 2 nA current over 1 h per 20 μL volume is at the order of 10^–6^ M (in terms of the transported charges), which is insignificant (orders magnitude lower) compared to bulk concentrations.

### Structure Characterizations

**Figure 2:**
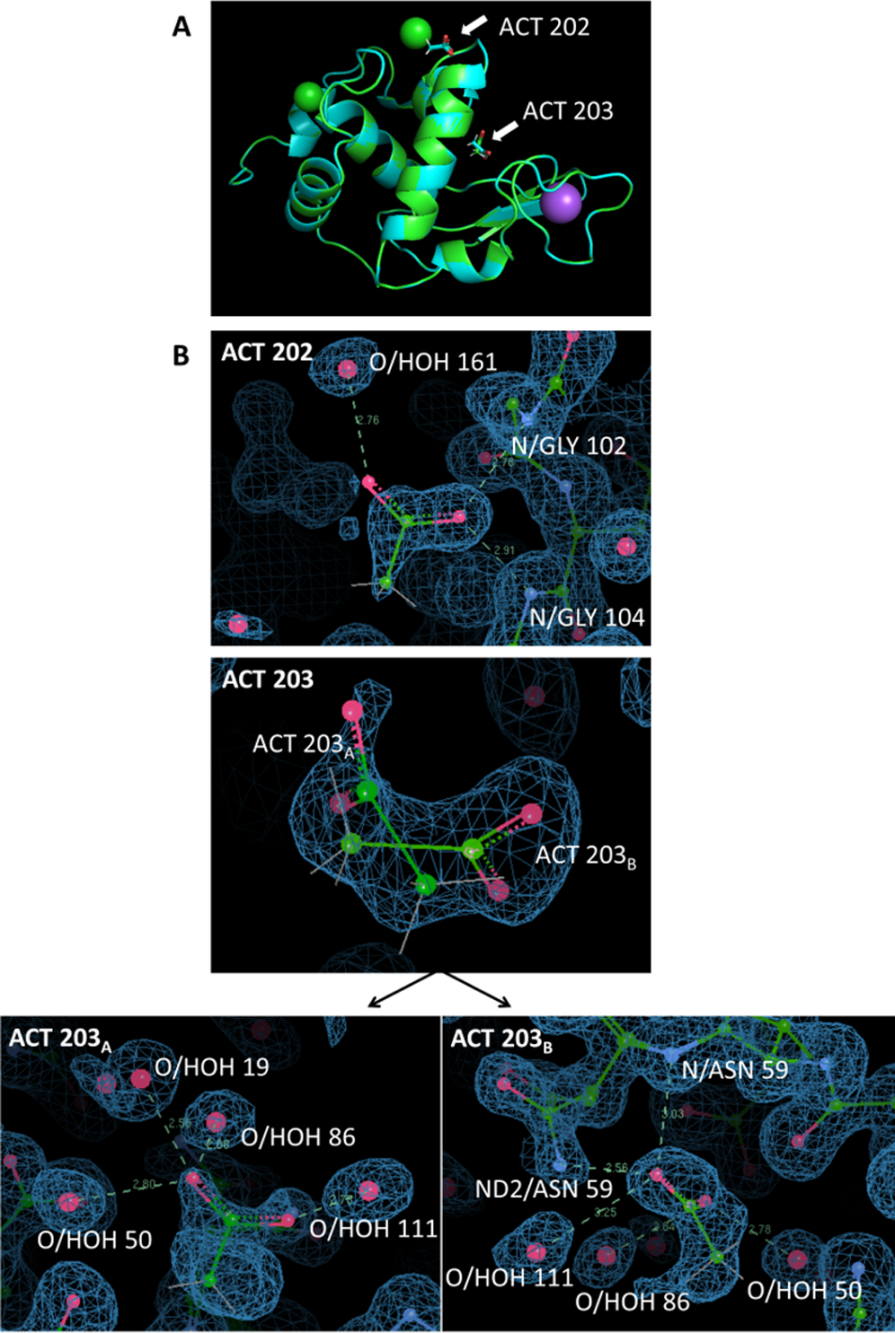
Structure characterizations. (A) The 3D structure including two chloride ions (green spheres), one sodium ion (purple), and two acetate ions (sticks highlighted by arrows). (B) 2Fo-Fc electron density maps at a contour level of 1σ for the acetate ions and their hydrogen-bonding interactions with surrounding residues/water molecules. ACT 202 has one single preferred orientation; ACT 203 has two orientations (ACT 203A and ACT 203B). Details of hydrogen-bonding interactions are listed in Table S4.

The NanoAC method synthesizes high-diffraction quality crystals with high consistency. Excluding those as controls and to survey sample preparation conditions, all five crystals grown under comparable NanoAC control parameters achieve atomic resolutions. Even for lysozyme that is widely used as a prototype, the consistently high quality, with crystals synthesized at room temperature over several hours, is impressive and approaches those grown in space under microgravity using the counter-diffusion method over days (resolution up to 0.94 Å, PDB ID: 1IEE).^56^ The space group P4_3_2_1_2 is consistent with literature (Tables S2 and S3). The resolution is up to 1.2 Å after refinement. The structure (PDB ID: 8F28) is most consistent with those structures that adopt a similar chemical environment (i.e., PDB: 1iee). Two acetate ligands are newly resolved in the electron density map, which improve the structure stability by forming multiple hydrogen bonding interactions with surrounding residues and water molecules (Figure 2B and Table S4). As shown in Figure 2, ACT 202—located at the edge of the protein—shows a single preferred orientation with the carboxyl group facing away from the structure. In contrast, ACT 203—located in the middle of the protein—has an occupancy for two ion orientations, or dual orientations in the crystal (referred to as ACT 203A/B). Besides the high atomic resolution for structure–function correlations, the NanoAC platform offers significant merits (over microgravity or EM) such as easy hardware accessibility for generalization and versatility for the incorporation with structure characterization tools. Detailed diffraction data and refinement results are listed in Tables S2 and S3. Corresponding crystal synthesis parameters such as nucleation and growth rates (explained next) are in Table S5. As controls, faster nucleation and growth rates lead to much lower diffraction quality (>3 Å resolution, Table S6) that is not further refined.

### Two Electroanalytical Signatures for Nucleation Kinetics: Changes in Current Amplitude and Noise Reduction

**Figure 3:**
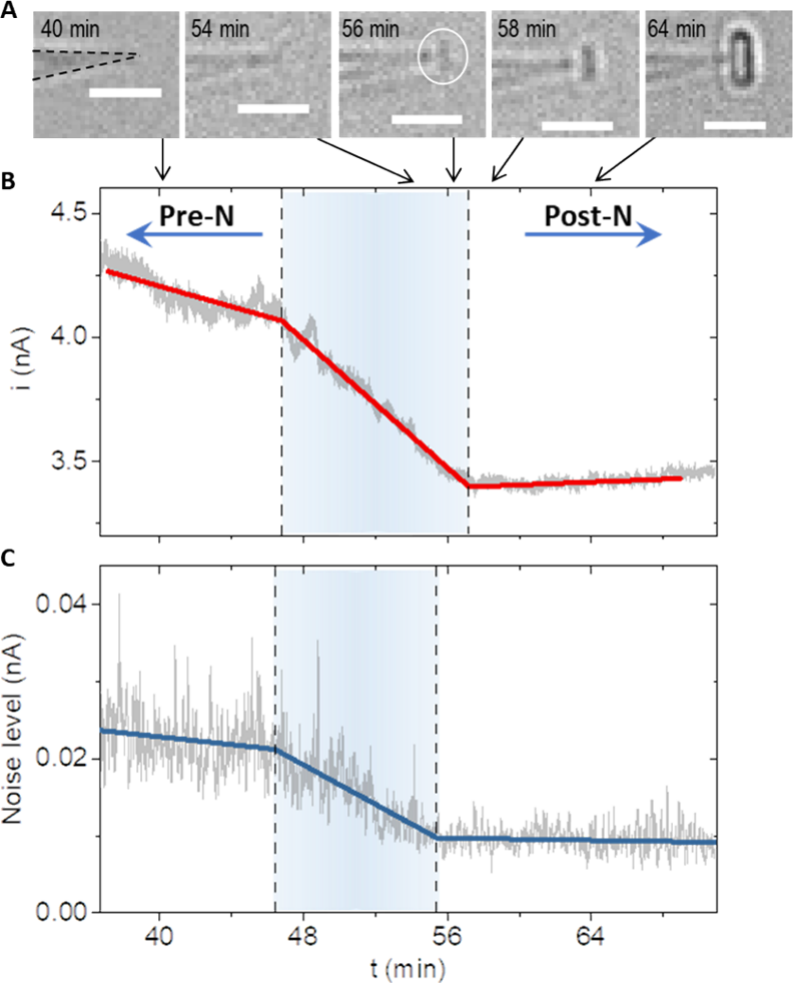
Electric current signatures for nucleation kinetics with a 40 nm-radius pipette. (A) Optical images at representative time points (scale bars, 4 μm). (B) Changes in ionic current and (C) changes in the noise level of the current during nucleation. The data sampling rate is 10 pts./second under +1.0 V. The 5 s or 50-point moving standard deviation (MSTD) of the current is used as noise level plotted in (C). Solid lines in red and blue are piecewise continuous linear regression of the unsmoothed data (gray). The dashed lines indicate the breakpoints in the piecewise model. The first breakpoint deviating from the pre-nucleation (Pre-N) baseline represents the start time point *t*_S_, and another breakpoint leading to the post-nucleation (post-N) baseline indicates the end time point *t*_E_. The baselines are established by sampling thousands of data points, i.e., 8 min or longer during pre-N and post-N periods. *P* < 0.0001 for all breakpoints.

By analyzing the current–time curve, the nucleation event that ultimately produces a single high diffraction-quality crystal is resolved. The data around nucleation is plotted in Figure 3 (other stages not included for clarity). Two electric signatures, the current amplitude and its noise level, are identified around the time when optically resolved lattice features emerge. Compared to the baseline current in the liquid domain stage (pre-N), a relatively abrupt and large current decrease precedes the optical features by seconds to minutes (Figure 3B). The noise level, characterized by the standard deviation of the current amplitude, reduces accordingly (Figure 3C).

**Figure 4:**
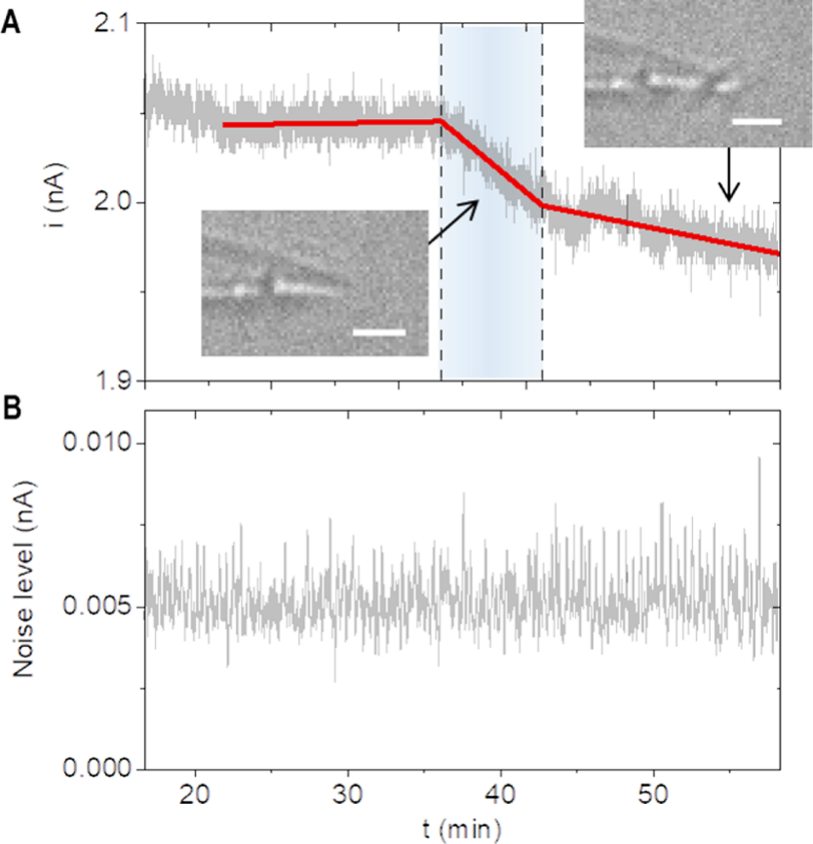
Electroanalytical signatures for nucleation kinetics with a 150 nm-radius pipette. (A) Changes in ionic current and (B) changes in noise level during nucleation. Inset: optical images at representative time points (scale bars, 4 μm). Data sampling rate is 10 pts./second under +0.2 V. Data treatment the same as described in Figure 3.

Both signatures are consistently observed in smaller nanopipettes (additional 40 nm ones in Figure S5). For larger nanopipettes, noise reduction is often absent and the changes in current amplitude are weaker (Figure 4 and Figure S6). The higher flux, however, generally induces domain formation faster and nucleation transition more likely compared to the smaller nanopipettes. Oscillative current variations are sometimes observed in smaller nanopipettes before nucleation or during crystal growth but are rare in larger nanopipettes (Figure S3A,B, pre-N; S3B, domain). Regardless of the common drift in baseline current, the distinct current oscillation, or the less-featured noise reduction, the electroanalytical signatures of nucleation can be consistently resolved and quantitated through piecewise regression as shown in those representative examples (occasionally data smoothing prior to fitting when excessive noise is present). Noteworthily, the reduction in noise levels is highly consistent as long as it is detectable. Therefore, combining the two electroanalytical signatures will effectively mitigate their respective limitations.

The physical origin of the noise and its changes are noteworthy to discuss. Similar current oscillations have been observed due to the nanoprecipitation of inorganic salts at conical nanopores by Siwy’s group.^57^ The curve shapes in those current oscillation events are reminiscent of the translocation of nanoparticles through conical nanopores explained by White’s group.^58^ Herein, the current signal is limited by either the liquid domain prior to nucleation or a solid-phase structure on the nanotip afterward. The higher noise levels during the pre-N period may result from the transient nuclei formations that are insufficient to overcome the thermodynamic energy barrier for further crystal growth.^12,24^ In addition to the dynamic structural changes of the pre-nucleation assemblies that are extremely challenging to characterize, their translocation at the nanotip region and the Ostwald ripening effects may also affect the transient current signals. Quantitative interpretation is beyond the scope of this work and will be pursued in future endeavors. Overall, the current change, noise reduction, together with optical observations, are established as three signal signatures that can corroborate and diagnose rare and challenging cases. Because nucleation by definition means the energy barrier is surpassed successfully, optically resolved lattice features emerge within seconds to minutes generally. Therefore, the third signature (optical observation) rejects the possibility of nucleation even if coincidental current changes and noise reduction are observed electrically. It is also worth mentioning that the absolute changes in current amplitude and noise level will depend on the physicochemical properties of sample:precipitant: the differences in the ion mobility between electrolyte ions and small molecules, as well as the differences in different biomacromolecules, may induce different absolute changes. For example, the high mobility and small size likely make the proton the main charge carrier in our earlier report on insulin crystallization under the pH gradient.^45^ For the multivariate gradients of salt and PEG:lysozyme, the exact mechanism is highly complex and remains to be determined. To speculate, the transport of Na^+^ and Cl^–^ should dominate the measured ionic current due to the higher mobility over the much larger lysozyme molecules. Those considerations, however, are not expected to affect the applications to unknown samples or using different nanopipettes. The signatures are self-calibrated within each individual measurement via the relative “abrupt” changes over the more gradual “continuous” baseline drift during the pre-N and post-N periods.

The nucleation rate (*V*_N_), determined with *t*_S_ and *t*_E_ as start and end points, is corroborated by the two electroanalytical signatures: 1.6 × 10^–3^ s^–1^ (46.83–57.27 min) from the current amplitude and 1.8 × 10^–3^ s^–1^ (46.40–55.42 min) from the noise level. The reproducibility is demonstrated by the comparable results from different nanopipettes in Tables S7 and S8. The nucleation rate, expressed in events per second, leading to the subsequent growth of a single entity, is in reasonable agreement with the lower end of nucleation rates reported in the literature (approx. 10^–3^–10^–1^ s^–1^, based on a 0.5 mL volume used).^55^ Considering that the uncertainties in the reported nucleation rates can vary by more than one order of magnitude, an independent method such as NanoAC is therefore highly desirable for comparison and validation. The onset *t*_S_ from electroanalytical signatures leads to optical changes consistently. This is believed significant for the detection of nucleation and early crystal growth, which are critical in controlling crystal quality.^59^

The structural evolution of spontaneous nucleation has been revealed by liquid-cell transmission EM.^59^ Optical imaging herein obviously lacks structural details but offers convenience and wide accessibility, without high-energy electron beams that might alter the molecular assembly dynamics. At the adopted potential and data sampling rates, superheating and bubble nucleation are unlikely but may warrant further study on its presence in the transient noise and current oscillation features during pre-nucleation (Figure S3A, pre-N; Figure S3B, domains, etc.).^12^

From the single entity point of view, successful nucleation should correspond to *THE* irreversible event that overcomes the energy barrier, as measured by the NanoAC. Other molecular assemblies form, grow, and redissolve, inducing the higher “current noise”. The pre-nucleation period, often not resolved in ensemble nucleation measurements, may account for the orders magnitude variations in the nucleation kinetics and the discrepancy with theoretical predictions. The electroanalytical signals depend on the position and size of the nuclei with respect to the transport-limiting region (radial and axis coordinates of the nanopore). Accordingly, those differences in the nucleation rates and induction time (data in Tables S7 and S8) are deemed acceptable considering the nanopipette heterogeneity, the stochastic nature of nucleation, and its sensitivity to subtle changes in reagent concentrations and temperature. The measured current in the multi-variant electrolyte systems herein is highly challenging to quantitate toward specific ionic species based on our earlier work and will be separately pursued.^34−39^

### Active Controls in the Crystal Habit and Monitoring Lattice Growth Rate at Single Entity Levels

**Figure 5:**
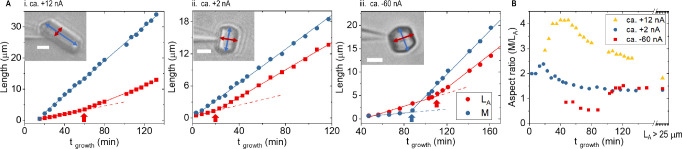
Crystal growth controlled at single-entity levels under different currents. (A) Growth kinetics. After nucleation initiated under +0.2 V, the potential was adjusted manually to about +1.0 V (Ai, ca. +12 nA), +0.2 V (Aii, ca. +2 nA), and −0.5 V (Aiii, ca. −60 nA) respectively. Data points were fitted at *R* > 0.99 by piecewise linear regression (breakpoint/s indicated by arrow). The corresponding current/potential–time curves are in Figure S7. Additional examples are in Figure S8. The directions of *M* (blue) and *L*_A_ (red) are labeled by the double-headed arrows in the brightfield images (scale bars, 4 μm). The lengths can only be measured after reaching two pixels or more (single pixel 250 nm; optical limit). (B) The comparison by aspect ratio. An aspect ratio of 2 (±1) at the start results from the two/one pixel resolution to measure the length growth, i.e., limited by the optical resolution.

The crystal habits are successfully controlled by tuning the flux around individual crystals under different applied potentials. The growth rates of three tetragonal lysozyme crystals are determined from the increase in length *L*_A_ and *M* shown in Figure 5A, from which the growth rates of (110) and (101) faces are calculated.^60^ The results are listed in Table S11, along with corresponding current–time data in Figure S7. The evolution of the three crystal habits is illustrated by the aspect ratios over time compared in Figure 5B. Under positive bias when both migration and diffusion increase supersaturation (Figure 5Ai and Aii), the length *M* increases linearly over time, with *G*_101_ at 2.3 and 1.5 nm/s, respectively. The *G*_110_ in the *L*_A_ direction displays two linear ranges, indicating two-stage growth with a slower rate initially followed by a faster growth rate (0.6–1.1 nm/s in Ai and 0.5–1.1 nm/s in Aii). Higher current (Ai vs Aii) sustains faster *G*_101_, but the impacts on *G*_110_ are insignificant. Under negative bias when migration opposes diffusion, the (110) face displays two-stage growth as well (0.6–1.5 nm/s in Aiii), and the late *G*_101_ remains unaffected at 2.2 nm/s. Surprisingly, the early *G*_101_, at 0.3 nm/s, is significantly suppressed in comparison to those under positive current. One should be aware that the exact quantitative rate or growth kinetics may vary due to the heterogeneity among different nanopipettes. Additional examples in Figure S8 under different current amplitudes show consistent features and attest the reproducibility, or the error range in repeats or ensemble measurements. Systematic measurements are underway to elucidate quantitative correlations between the flux and growth rate. Overall, the reproducibility of this method is markedly high, as evidenced by the attainment of atomic resolution in all five crystals utilized for crystallography, and comparable nucleation rates and growth rates (Tables S7–S10).

A two-stage growth kinetics, in particular a distinctively slower early growth rate, is unique to the NanoAC method to the best of our knowledge. The faster growth rate during the later stage is on par with the literature values, where the growth kinetics is governed by the intrinsic thermodynamics of the chemical system, not NanoAC controls, i.e., energy differences of solubilized building blocks (individual lysozyme molecules and/or their oligomers) versus those assembled on the crystal lattice (and different facets). Mechanistically, the effective electric field in the sample solution will decrease with the crystal growth because the potential drops mostly in the nanotip region, increasingly occluded by the crystal during the growth. Consequently, the migration effect decreases and ultimately the crystal growth will be similar to bulk/ensemble growth driven by diffusion. The explanation is further supported by the converging trend in aspect ratios when the crystals are larger, with length *L*_A_ > 25 μm over about 6 h (constant late growth of *G*_110_ and *G*_101_). Longer growth, however, can be affected by evaporation-induced increase in supersaturation.^61^

The slower initial growth, inaccessible in classic measurements, is attributed to nano-transport effects. A slower kinetics is counterintuitive because facilitated mass transport is a key merit of nanoelectrodes, in other words, higher influx of lysozyme via radial diffusion *AND* migration contributions are anticipated under a positive current/electric field. Our hypothesis is that rotational diffusion is reduced at the viscous liquid domain and under high flux, which inhibits the orientational adjustment during the assembly of individual building blocks and thus slows down the early growth. A similar concept has been proposed to explain the kinetic roughing of lysozyme crystals at very high supersaturations.^62^ Orientation alignment effects could also account for the more effective modulation in the M-direction growth under NanoAC, in reference to literature where crystal growth is closely associated with the volume fraction of bulkier octamers, as the building unit for the (110) face.^63,64^

Two-stage growth kinetics can also be observed without applied potential (Figure S9), where the streaming current is observed due to diffusion driven by concentration gradients. The result strongly suggests that the transport-limited growth by the nanotip-localized nucleation itself is adequate to slow down the earlier crystal growth, whereas the applied electric field further modulates the growth rate with greater flexibility. For example, delays in growth after nucleation (Figure 5C and 5A versus Figure 5B) are observed when the potential is adjusted. While classic electrical double layer (EDL) charging and nanoscale EDL hysteresis associated with potential changes can account for some early current disruptions such as exponential decays in current spikes (Figure S7B, near time zero of growth), detailed mechanism and quantitative correlations between growth rate and control parameters remain to be established. Furthermore, crystal morphology also correlates with nucleation and growth rate.^65^ A high supersaturation can induce uncontrolled nucleation, optical defects, or kinetic roughing. The crystal morphology tends to deteriorate over time under negative flux presumably due to the faster late growth rate.

## Conclusions

To summarize, the NanoAC method synthesizes high-quality crystals in high consistency with unprecedented single-entity controls. Electroanalytical and optical signatures from real-time monitoring of the whole nucleation and crystal growth processes are established as quantifiable feedbacks for the controls and correlation with the crystal quality. Besides the more obvious successes in controlling diffraction quality and crystal habits, nucleation and growth rates from the continuous monitoring of individual phase transitions provide the much-needed links for the often-disconnected theoretical predictions and ensemble experiments. As a general guide for practice, the supersaturation for crystallization can be achieved in a wide range of bulk concentrations of precipitants and sample-of-interest by adjusting their concentrations and gradients across the nanotip and tunable by the external potential. To obtain a desired crystal habit or to achieve certain physiochemical properties, adjustments in ion transport during the growth can be achieved conveniently by programming the potential waveform that differentiates the growth rates of different lattices over different stages.

## Supporting Information Available

Experimental and data processing details; conductivity size measurements of pipettes; X-ray diffraction data, structural refinement results, and the corresponding NanoAC control parameters; bonding interactions of acetate ions with water molecules and neighboring residues; mehod reproducibility; additional nucleation and crystal growth results and analyses; current/potential–time profiles corresponding to the crystal growth; crystal growth and corresponding current/potential–time curves under less electric field manipulation (PDF)
Crystal growth video 1 (AVI)
Crystal growth video 2 (AVI)
Crystal growth video 3 (AVI)

## Supplementary Material

ac3c00175_si_001.pdf

ac3c00175_si_002.avi

ac3c00175_si_003.avi

ac3c00175_si_004.avi
