# Supplementary material for: A Single-Entity Method for Actively Controlled Nucleation and High-Quality Protein Crystal Synthesis
Source: Anal Chem. 2023 May 27;95(25):9462–70. doi: 10.1021/acs.analchem.3c00175 (PMC10308327; doi:10.1021/acs.analchem.3c00175)
Supplement: Supplementary file 1 — ac3c00175_si_001.pdf [file ac3c00175_si_001.pdf]

Supplementary Materials for

A Single-Entity Method for Actively Controlled Nucleation and High-Quality Protein Crystal Synthesis

Ruoyu Yang, Maksim Kvetny, Warren Brown, Edwin N Ogbonna, Gangli Wang*

Department of chemistry, Georgia State University, Atlanta, GA, 30302

**Correspondence should be addressed to glwang@gsu.edu*

This PDF file includes:

Additional details in materials and methods
- Chemicals, materials, and instrumentation
- Crystallization procedures
- Growth rate measurements
- Electroanalytical data processing
- Crystallography and data processing

Table S1. Conductivity size measurements of nine 150-nm-radius pipettes.
Table S2. X-ray diffraction data from five crystals synthesized under optimized NanoAC controls.
Table S3. Structure refinement results.
Table S4. Lists of bonding interactions of acetate ions with water molecules and neighboring residues.
Table S5. The NanoAC control parameters for the syntheses of the crystals in Table S1.
Table S6. The NanoAC control parameters result in lower diffraction quality (3~4 Å).
Table S7. Nucleation rates determined by current decrease ($V_{N,C}$) and noise reduction ($V_{N,N}$) from four 40-nm-radius pipettes.
Table S8. Nucleation rate determined by current decrease ($V_{N,C}$) from six 150-nm-radius pipettes.
Table S9. Growth rates of face (110) from four 40-nm-radius pipettes.
Table S10. Growth rates of face (110) from nine 150-nm-radius pipettes.
Table S11. The growth kinetics of individual crystals under varied electric current controls.

Figure S1. Current-time trace under different potentials to induce the domain formation using a 150-nm-radius pipette.
Figure S2. Current-time traces from three pipettes with different reagent gradients across the nanotip.
Figure S3. Electric current features of three distinct phase transition periods from two 40-nm-radius pipettes.
Figure S4. Electric current features of three distinct phase transition periods from two 150-nm-radius pipettes.
Figure S5. Electric signatures for nucleation kinetics from two 40-nm-radius nanopipettes.
Figure S6. Electric signatures for nucleation kinetics from one 150-nm-radius nanopipette.
Figure S7. Current-time and potential-time profiles corresponding to the crystal growth in Fig. 5.
Figure S8. Crystal growth controlled at single-entity levels under different current amplitudes.
Figure S9. Crystal growth and corresponding current/potential-time curves under less electric field manipulation.

Other supplementary material for this manuscript includes:

Movies S1-S3 of crystal growth

Captions:

S1. Crystal growth video 1 (10micronScale, 60minDuration)

S2. Crystal growth video 2 (10micronScale, 110minDuration)

S3. Crystal growth video 3 (10micronScale, 120minDuration)

Materials and methods

Chemicals and materials

All the chemicals were used as received without further purification, including Hen egg white lysozyme (HEWL, ≥ 90%, Sigma-Aldrich), sodium acetate anhydrous (fused, Fisher Scientific), sodium chloride (crystalline, Fisher Chemical). α,ω-Dicarboxyl polyethylene glycol (HOOC-PEG-COOH, MW: 3.5 kDa, ≈96.8%, Advanced BioChemicals). All solutions were filtered with a 0.45-μm PES syringe filter (Celltreat) prior to usage.

Nanopipette fabrication, characterization and loading

Nanopipettes were fabricated from quartz capillaries (O.D.: 1.0 mm, I.D.: 0.70 mm, 7.5 cm length, QF100-70-7.5, Sutter Instrument Co.) using a P-2000 laser puller (Sutter Instrument Co.). The pulling parameters for 40-nm-radius nanopipettes are: Heat: 700, Filament: 4, Vellocity:60, Delay:150, Pull: 120; for 150-nm-radius nanopipettes: line 1: Heat: 700, Filament: 4, Vellocity:55, Delay:180, Pull: 80, line 2: Heat: 700, Filament: 4, Vellocity:60, Delay:150, Pull: 120. The nanopipettes were backloaded using an in-house-made micro-injector constructed with a syringe and PTFE tubing. Centrifugation at 5000 rpm for 15 min was used after the manual loading to ensure the filling of the solution into the nanotip. Because individual nanopipettes can be heterogeneous in geometry and surface charge distribution, conductivity characterization is adopted for size characterization, which is routine in literature, rather than the sacrificial techniques such as scanning electron microscopy (SEM). The corroboration between the conductivity measurements with 1 M KCl solution and SEM is in ref. 45 among other literature reporting similar strategies.

The conductivity size characterization of a conical nanopore is summarized in a recent review (ref. 44):

$$R_{cone} = \frac{1}{\pi \Lambda r tan\theta} + \frac{1}{4\varepsilon R}$$

Here, R_{cone} is the Ohmic resistance of the nanopore; Λ is electrical conductivity of the electrolyte; r is the radius of a nanopipette; θ is the half cone angle (Scheme S1). A 1 M KCl solution is generally used in conductivity size characterizations: linear ohmic current-potential curve confirms the surface effects being insignificant and allows for the volumetric resistance calculation. Access resistance $\frac{1}{4\varepsilon R}$ was not considered at this concentration. The θ was determined to be 3.5° by SEM and assumed consistent. The Λ was 10.9 S/m for 1 M KCl solution. Conductivity size measurements for nine 150-nm-radius pipettes were summarized in Table 1.

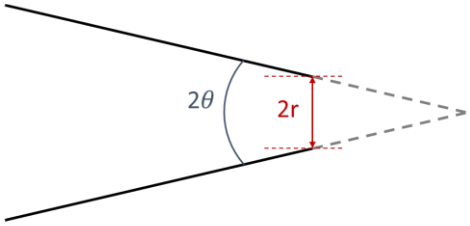

Scheme S1 The geometry of a conical nanopore.

Details for crystallization

A 3D printed ABS chamber (25×50 mm^2) was used, consisting of a 1-mm-depth well, a 1-mm slit channel on the top that accommodates pipette and a narrow channel at the bottom for a Ag/AgCl WE. Once the WE was placed, a plastic coverslip was attached to the bottom of the well by vacuum grease. Then, a pipette loaded with the precipitant solution was inserted into the well through the slit channel, and 10-20 μL mother solution was added until the WE and pipette tip were immerged. After that, the chamber was sealed with a coverslip on the top to reduce evaporation.

The crystallization solution was freshly made by mixing equal volumes of the protein stock solution and a 1.1 M NaCl and centrifuged at 15,000 rpm for 10 min. The choice of 1.1 M NaCl to approach supersaturation is based on the screening using 1.0 M, 1.1 M and 1.2 M NaCl. By using the 1.0 M NaCl instead of 1.1 M to prepare the sample for nanopipette experiments, nucleation is not observed within one day under otherwise comparable conditions in NanoAC controlled crystallization. In comparison as summarized in Table S7 and S8, the induction time, starting from the application of the positive potential till the start of nucleation t_S, is much faster within several hours.

Growth rate measurement

A series of time-lapsed images were taken during the growth of individual crystals. Image size/scale was calibrated with a Stage Micrometer KR868 (Klarmann Ruling) and analyzed with ImageJ. The crystal sizes in the lengths of M and L_A (Fig. 1 and Fig. 3) were used to calculate the crystal growth rates of the (101) and (110) faces of the tetragonal lysozyme crystals as described by Durbin and Feher [36]

$$G_{110} = 0.50\, d_{L_A}/d_t$$

$$G_{101} = 0.45\, d_M/d_t + 0.30\, G_{110}$$

Electroanalytical data processing

The electric current over the whole phase transition was generally recorded at ten points per second rate (can be faster or slower if needed) and used directly without denoising or outlier removal. The noise level is represented by 5-second (50-point) moving standard deviation of the raw current. To resolve the transition point times, continuous piecewise linear regression with two breakpoints was performed using Origin. With enough sampling points for the pre-N and post-N baselines, i.e., 8 min, the breakpoints can be determined with $P < 0.0001$. If spikes/oscillations were continuously presented in the current (Fig. S5B, Noise level), data was smoothed by 20% percentile filter using 1% points prior to piecewise fitting.

Crystallography and data processing

Crystals were synthesized one at a time. After the size reaches about 70-90 µm of length in L_A, the pipette with a crystal still attached on tip was removed from the sample droplet and quickly inserted in LV CryoOil (MiTeGen) for 20-30 s prior to flash-cooling in liquid nitrogen. Ten tetragonal lysozyme crystals were harvested in this manner for X-ray crystallography. Five of them grown at 1.8±0.9 nm/s generated high diffraction quality, and five of them grown at a higher growth rate showed poor diffraction. The results are summarized in Tables S2 and S3.

X-ray diffraction data were collected at 100 K from beamline 8.2.1 (λ=1.0000 Å) at the Advanced Light Source (ALS, Berkeley, CA). Diffraction data were indexed, integrated and scaled using HKL-2000 program package based on a Chi^2=0.5 cutoff. Molecular replacement and refinement of the reduced scalepack files were performed using Phenix 1.20 and Coot 0.9.2. X-ray data collection and refinement statistics are listed in Table S3. Ramachandran favored 98.43%, allowed 1.57%, outliers 0.00%.

Table S1. Conductivity size measurements of nine 150-nm-radius pipettes. Nine pipettes were measured with 1 M KCl solution inside and outside of the nanopore. R_{cone} is the Ohmic resistance of the nanopore; r is the radius of a nanopipette; θ is the half cone angle (Scheme S1) at 3.5°; Λ is electrical conductivity of the 1 M KCl solution at 10.9 S/m.

Pipet #	$i_{-50\ mV}$ (A)	$i_{50\ mV}$ (A)	R_{cone} (1/S)	r (nm)
1	2.57E-09	3.63E-08	2.96E+06	161
2	2.08E-09	3.14E-08	3.42E+06	140
3	3.21E-09	3.84E-08	2.84E+06	168
4	3.46E-09	4.33E-08	2.51E+06	190
5	3.58E-09	3.56E-08	3.13E+06	153
6	2.23E-09	4.04E-08	2.62E+06	182
7	3.00E-09	4.15E-08	2.60E+06	184
8	2.05E-09	3.45E-08	3.08E+06	155
9	2.08E-09	2.82E-08	3.83E+06	125
Average				162
SD				22

Table S2. X-ray diffraction data from five crystals synthesized under optimized NanoAC controls. Crystal 4 here is crystal 1 in Table S8 and S10. Crystal 5 here corresponds to the current and growth in Figure S9B.

**Data collection**	Crystal 1	Crystal 2	Crystal 3	Crystal 4	Crystal 5
Space group	$P4_32_12$	$P4_32_12$	$P4_32_12$	$P4_32_12$	$P4_32_12$
Cell dimensions					
a, *b*, *c* (Å)	78.59, 78.59, 36.82	78.75, 78.75, 36.92	78.11, 78.11, 36.85	78.11, 78.11, 36.89	78.61, 78.61, 36.94
a, b, g (°)	90, 90, 90	90, 90, 90	90, 90, 90	90, 90, 90	90, 90, 90
Resolution (Å)	50-1.20 (1.22-1.20)	50-1.27 (1.29-1.27)	50-1.30 (1.32-1.30)	50-1.34 (1.36-1.34)	50-1.32 (1.34-1.32)
R_{meas}	0.079 (0.346)	0.067 (0.376)	0.070 (0.431)	0.076 (0.511)	0.065 (0.455)
I / s*I*	46.9 (2.7)	61.9 (4.3)	67.5 (2.7)	55.3 (3.4)	55.3 (3.3)
Completeness (%)	96.4% (71.2%)	99.7% (99.3%)	99.4% (91.9%)	99.9% (100.0%)	98.5% (76.0%)
Redundancy	7.0 (2.0)	11.0 (5.5)	11.9 (6.0)	10.2 (7.4)	8.0 (5.2)

Table S3. Structure refinement results of crystal 1 in Table S2.

**Refinement***	A	B	AB
Resolution (Å)	30.69-1.20		
No. reflections	35226		
R_{work} / R_{free}	0.1430/0.1715	0.1419/0.1691	0.1423/0.1695
No. atoms			
Protein	1111		
Ligand/ion	5 **		
Water	165		
B-factors			
Protein	13.40		
Ligand/ion	22.34	21.97	21.57
Water	28.40	28.36	28.38
R.m.s. deviations			
Bond lengths (Å)	0.006		
Bond angles (°)	0.867	0.864	0.870

* Refinement results that kept one orientation (A and B) and two orientations (AB) for ACT 203 were compared. Only results that differ from orientation A were shown.

** Structure includes one Na ion, two Cl ions, and two ACT ligands. ACT 203 has one or two orientations.

Table S4. Lists of bonding interactions of acetate ions with water molecules and neighboring residues. The residue number is from the peptide sequence, and the number for water molecules is generated by software during refinement.

**Acetate ion**	**H-Bonding interactions**	**Distance (Å)**
ACT 202	$_{Y}$C=O --- O/HOH 161	2.7
	$_{X}$C=O --- N/GLY 102	2.8
	$_{X}$C=O --- N/GLY 104	2.9
ACT 203_{A}	$_{X}$C=O --- O/HOH 19	2.6
	$_{X}$C=O --- O/HOH 50	2.8
	$_{X}$C=O --- O/HOH 86	2.9
	$_{Y}$C=O --- O/HOH 111	2.8
ACT 203_{B}	$_{X}$C=O --- N/ASN 59	3.0
	$_{X}$C=O --- ND2/ASN 59	2.6
	$_{Y}$C=O --- O/HOH 50	2.8
	$_{Y}$C=O --- O/HOH 86	2.7
	$_{X}$C=O --- O/HOH 111	3.3

Table S5. The NanoAC control parameters for the syntheses of the crystals in Table S2. Crystal 4 here is crystal 1 in Table S8 and S10. Crystal 5 here corresponds to the current and growth in Figure S9B.

Parameter	Crystal 1	Crystal 2	Crystal 3	Crystal 4	Crystal 5
Nanotip radius (nm)	40	40	40	150	150
Post-Nucleation current, i (nA)	2	3	4	3	-2
Nucleation rate, $V_{N,C}$ ($\times 10^{-3}$ s^{-1})	/	2.8	1.0	2.7	1.2
Growth rate, G_{110} (nm/s)	1.7	1.0	0.60	0.66	0.50

* Nucleation rate is calculated from current decrease. Nucleation rate for crystal 1 was not recorded thus not listed. The growth rates were measured during the linear growth period when the length of L_A grow from about 3 μm to 20 μm or larger.

Table S6. The NanoAC control parameters result in lower diffraction quality (3~4 Å).

Parameter	Crystal 6	Crystal 7	Crystal 8	Crystal 9	Crystal 10
Nanotip radius (nm)	40	40	40	150	150
Post-Nucleation current, i (nA)	4	9	2	17	5
Nucleation rate, $V_{N,C}$ ($\times 10^{-3}$ s^{-1})	5.5	100	83	5.0	/
Growth rate, G_{110} (nm/s)	8.5	11	6.5	4.2	3.4

* Nucleation rate for crystal 10 were not recorded thus not listed. The growth rates were measured during the period when the length of L_A grow from about 70 μm to 90 μm.

Table S7. Nucleation rates determined by current decrease ($V_{N,C}$) and noise reduction ($V_{N,N}$) from four 40-nm-radius pipettes. Nucleation rate is calculated as the reciprocal of the time difference (t_{End} - t_{Start}). The starting time $t_{S,C}$ and $t_{S,N}$ correspond to the induction time after the driving potential is applied. Crystal 1 here is used for Figure 1A and Figure 3. Crystal 1-4 correspond to crystal 1-4 in Table S9. These data were collected using a previous generation cell (ref. 42) where evaporation causes the supersaturation to increase and corresponding faster phase transitions.

Crystal	$t_{S,C}$ (s)	$t_{E,C}$ (s)	$t_{S,N}$ (s)	$t_{E,N}$ (s)	$V_{N,C}$ (10^{-3} s^{-1})	$V_{N,N}$ (10^{-3} s^{-1})
1	2790	3468	2798	3362	1.6	1.8
2	2324	2549	1757	2565	4.4	1.2
3	2240	2369	2345	2451	7.7	9.4
4	2044	2195	1984	2244	6.6	3.9
Mean	$2.3_5\times10^3$	$2.6_5\times10^3$	$2.2_2\times10^3$	$2.6_6\times10^3$	5.1	4.1
SD	$0.3_2\times10^3$	$0.5_7\times10^3$	$0.4_5\times10^3$	$0.4_9\times10^3$	$2._7$	$3._7$

Table S8. Nucleation rate determined by current decrease ($V_{N,C}$) from six 150-nm-radius pipettes. Note as stated in the main text, the signature of noise level can only be reliably obtained using smaller nanopipettes. Crystal 4 here is crystal 4 in Table S2 and S5 (crystallography). Crystal 5 here is the crystal grown at ca. +2 nA in Figure 5 (growth habit). Crystal 1-6 correspond to crystal 1-6 in Table S10.

Crystal	$t_{S,C}$ (s)	$t_{E,C}$ (s)	$V_{N,C}$ (10^{-3} s^{-1})
1	14361	16180	0.6
2	7795	8123	3.0
3	9315	9680	2.7
4	11139	11557	2.4
5	12172	12709	1.9
6	9104	9525	2.4
Mean	$1.0_6 \times 10^4$	$1.1_3 \times 10^4$	2.5
SD	$0.2_4 \times 10^4$	$0.2_9 \times 10^4$	0.4

Table S9. Growth rates of face (110) from four 40-nm-radius pipettes. The growth rates were measured during the linear growth period when the length of L_A grow from about 3 µm to 20 µm or larger. Crystal 1 here is used for Figure 3. Crystal 1-4 correspond to crystal 1-4 in Table S7.

Crystal	Grow rate, G_{110} (nm/s)
1	1.4
2	1.9
3	1.8
4	2.0
Mean	1.8
SD	0.3

Table S10. Growth rates of face (110) from nine 150-nm-radius pipettes. The growth rates were measured during the linear growth period when the length of L_A grow from about 3 µm to 20 µm or larger. Crystal 4 here is crystal 4 in Table S2 and S5 (crystallography). Crystal 5 here is the crystal grown at ca. +2 nA in Figure 5 (growth habit). Crystal 1-6 correspond to crystal 1-6 in Table S8.

Crystal	Grow rate, G_{110} (nm/s)
1	0.9_7
2	1.1
3	0.8_6
4	0.6_6
5	1.1
6	0.8_9
7	0.8_8
8	0.9_0
9	0.8_7
Mean	0.9
SD	0.1_2

Table S11. The growth kinetics of individual crystals under varied electric current controls (data in Figure 5A-C).

Current	L_A, early (nm/s)	L_A, late (nm/s)	t_{bp} (min)	M, early (nm/s)	M, late (nm/s)	t_{bp} (min)
**Ca. +12 nA**	1.1±0.1*	$2.1±0.0_3$	58±2	$4.6±0.0_4$		/
**Ca. +2 nA**	0.9±0.2	$2.1±0.0_2$	20±2	$2.6±0.0_2$		/
**Ca. -60 nA**	1.2±0.1	3.0±0.1	85±2	0.3±0.2	3.9±0.1	110±3

t_{bp}: the breakpoint from piecewise linear regression fitting.

*Note that, the standard deviation in the table was from the piecewise linear regression fitting, not to demonstrate reproducibility.

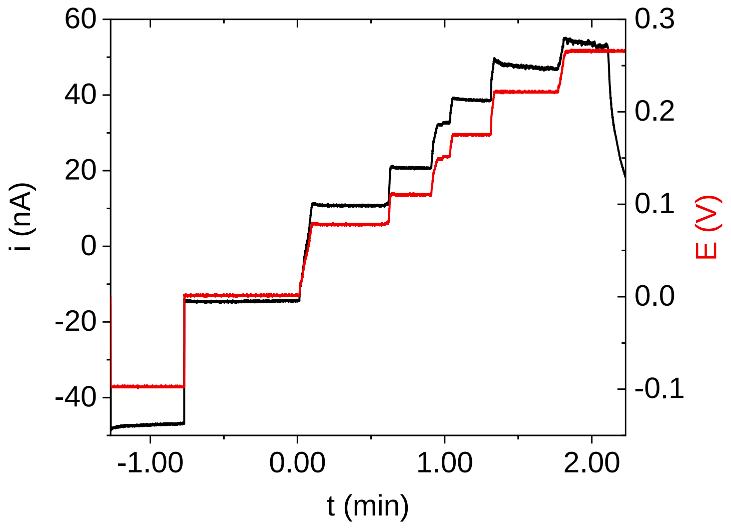

Figure S1. Current-time trace under different potentials to induce the domain formation using a 150-nm-radius pipette. A -0.1 V precondition potential was applied followed by stepwise increments (red curve, right y-axis). Domain formation was induced when ion flux passed a certain threshold (~40 nA for this pipette) indicated by a drastic current drop at around 2 mins. The current was constant at less positive potentials. The streaming current at zero voltage corresponds to the diffusion driven by the concentration gradients.

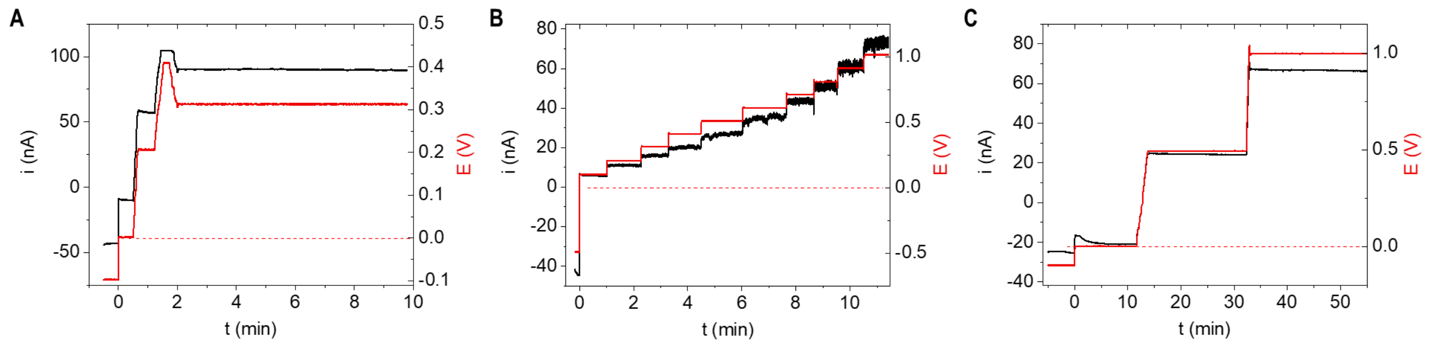

Figure S2. Current-time traces from three pipettes with different reagent gradients across the nanotip. (A) without NaCl gradient; (B) without COOH-PEG-COOH in precipitant solution, and (C) without protein in droplet solution. Detailed crystallization conditions were: (A) 25 mg/mL lysozyme, 0.6 M NaCl in droplet solution; 0.6 M NaCl, 10%(v/v) COOH-PEG-COOH in precipitant solution. (B) 25 mg/mL lysozyme, 0.6 M NaCl in droplet solution, 2 M NaCl in precipitant solution. (C) 0.6 M NaCl (no lysozyme) in droplet solution, 2 M NaCl, 10%(v/v) COOH-PEG-COOH in precipitant solution. Precondition potential at -0.1 V was applied, followed by several potential adjustments. Current was near constant in those three conditions, and no domain formation was optically resolved. A and B were from two ca. 150-nm-radius pipettes and C was from one ca. 40-nm-radius pipette.

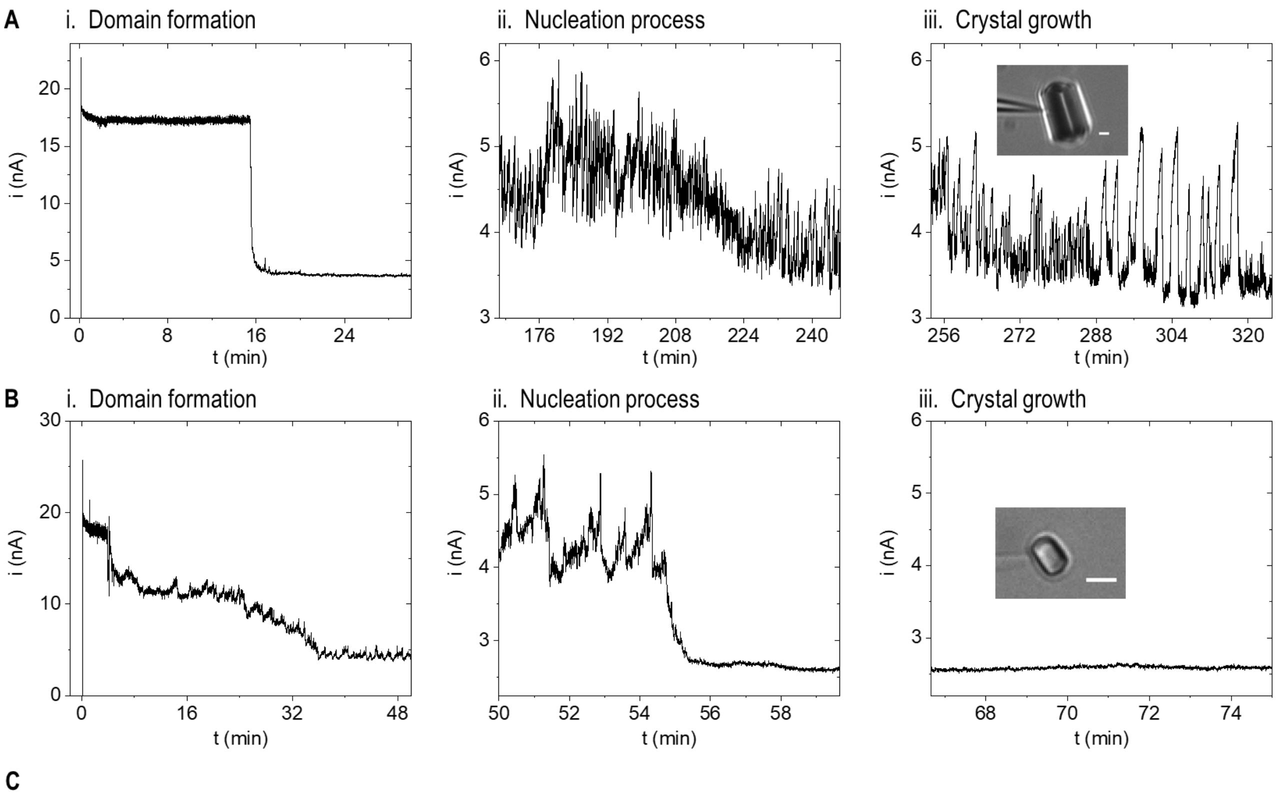

Figure S3. Electric current features of three distinct phase transition periods from two ca. 40-nm-radius pipettes. The potential bias (reference/counter electrode inside the nanopipette) was switched from -0.2 V to +1 V at time zero and held constant afterwards. Insert: representative optical images showing pristine morphology during crystal growth, taken at 380 min in (**A**) and 64 min in (**B**). Scale bars are 4 µm. Note current scales can be different in different panels.

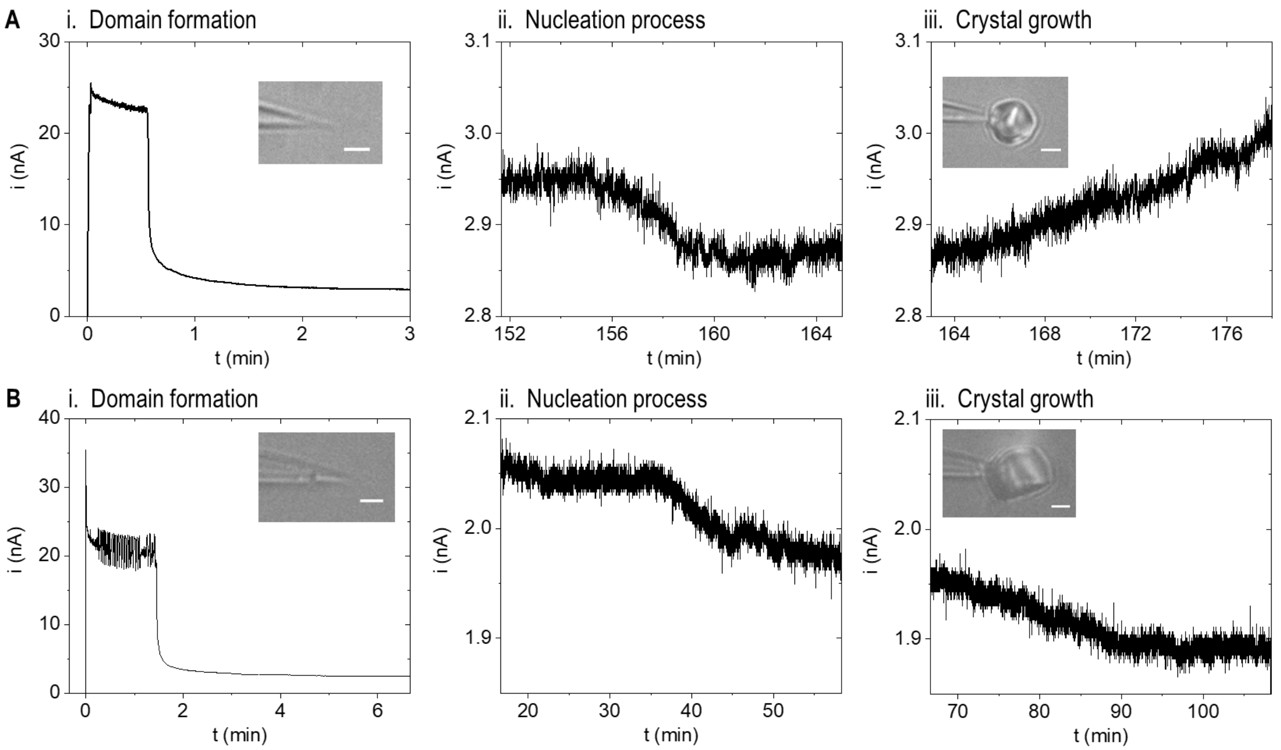

Figure S4. Electric current features of three distinct phase transition periods from two ca. 150-nm-radius pipettes. The potential bias (reference/counter electrode inside the nanopipette) was switched from -0.1 V to +0.25 V at time zero and held constant afterwards. Insert: representative optical images showing pristine morphology during crystal growth, taken at 235 min in (**A**) and 173 min in (**B**). Scale bars are 4 µm. Note current scales can be different in different panels.

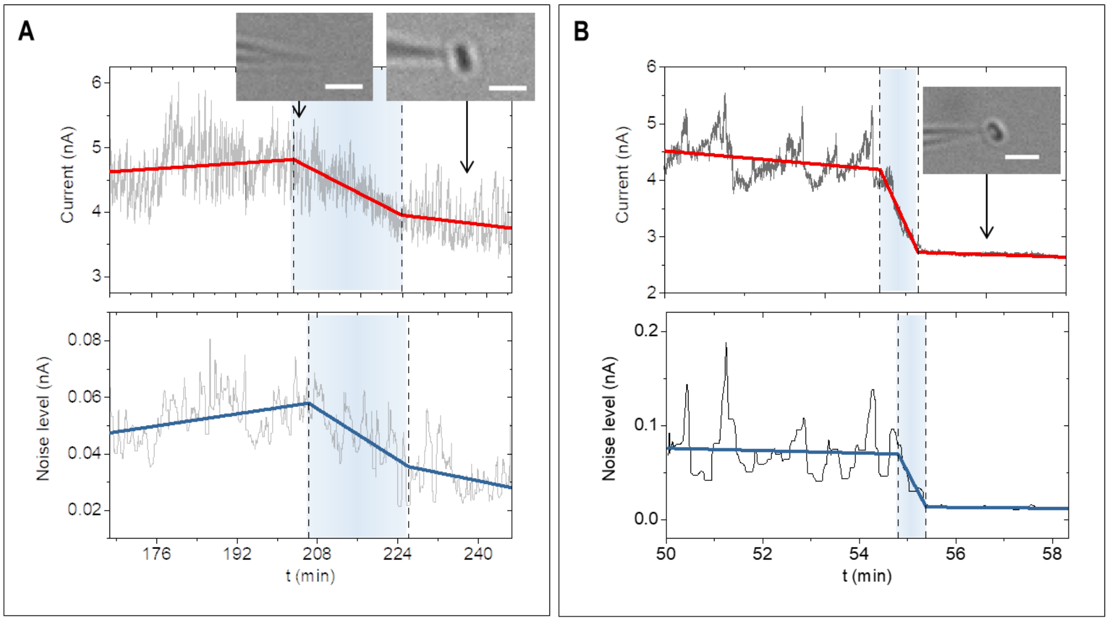

Figure S5. Electroanalytical signatures for nucleation kinetics from two ca. 40-nm-radius nanopipettes. Data sampling rate is 10 pts/second under +1.0 V. Five-second or fifty-point moving standard deviation (MSTD) of the current is used as noise level. Solid thick lines are piecewise continuous linear regression of the unsmoothed data (gray) except the noise level in B, that was smoothed by 20% percentile filter using 1% points. The vertical dashed lines indicate the breakpoints in piecewise model. The first breakpoint deviating from the pre-nucleation (Pre-N) baseline represents the start time point t_S; and another breakpoint leading to the post-nucleation (post-N) baseline signals the end time point t_E. The pre-/post-N baselines are established by sampling thousands data points, i.e. 8 mins or longer. P<0.0001 for all breakpoints. Insert: optical images at representative time points during nucleation. Scale bars are 4 μm. Note current scales can be different in different panels.

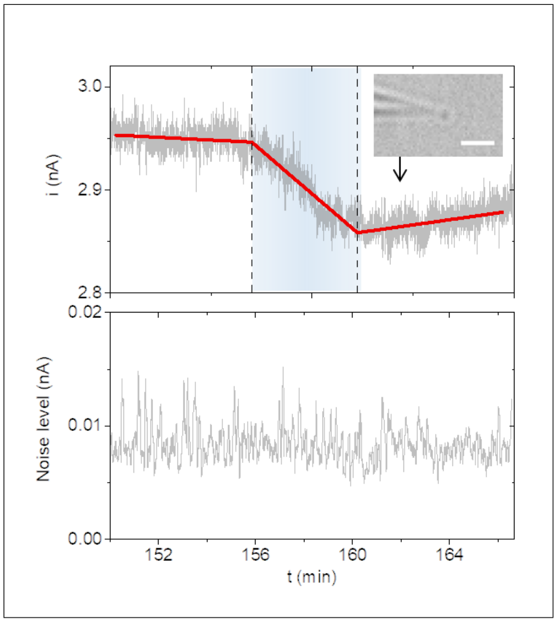

Figure S6. Electroanalytical signatures for nucleation kinetics from one ca. 150-nm-radius nanopipette. Data sampling rate is 10 pts/second under +0.25 V. Five-second or fifty-point moving standard deviation (MSTD) of the current is used as noise level. Solid thick lines are piecewise continuous linear regression of the unsmoothed data (grey). The vertical dashed lines indicate the breakpoints in piecewise model. The first breakpoint deviating from the pre-nucleation (Pre-N) baseline represents the start time point t_S; and another breakpoint leading to the post-nucleation (post-N) baseline signals the end time point t_E. The pre-/post-N baselines are established by sampling thousands data points, i.e. 8 mins or longer. Lack of noise level changes was observed. P<0.0001 for all breakpoints. Insert: optical images at representative time points during nucleation. Scale bars are 4 µm. Note current scales can be different in different panels.

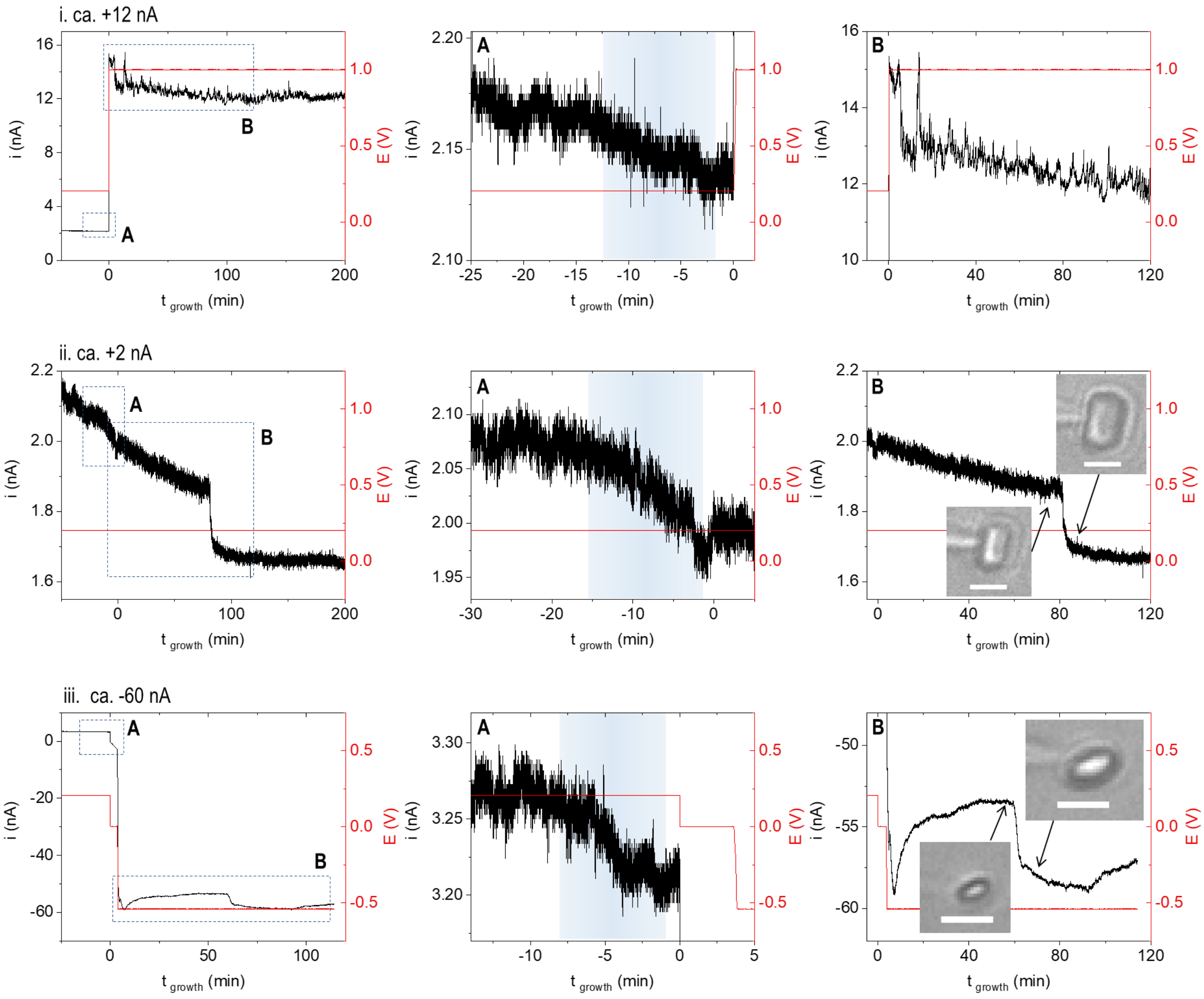

Figure S7. Current-time and potential-time profiles corresponding to the crystal growth in Fig. 5. Time zero is set after the nucleation process initiated under ca. + 0.25 V. The crystal growth is actively controlled under the current of ca. + 12 nA (**i**), ca. + 2 nA (**ii**), and ca. - 60 nA (**iii**) by adjusting the applied potential after nucleation signatures are detected (Blue shade period in zoom-in panel **A**). Actively controlled growth is in zoom-in panel **B** in which occasional abrupt current changes are observed. Optical images prior to and after the current changes are inserted to demonstrate that no obvious morphology changes are associated with those abrupt current changes. Scale bars are 4 μm.

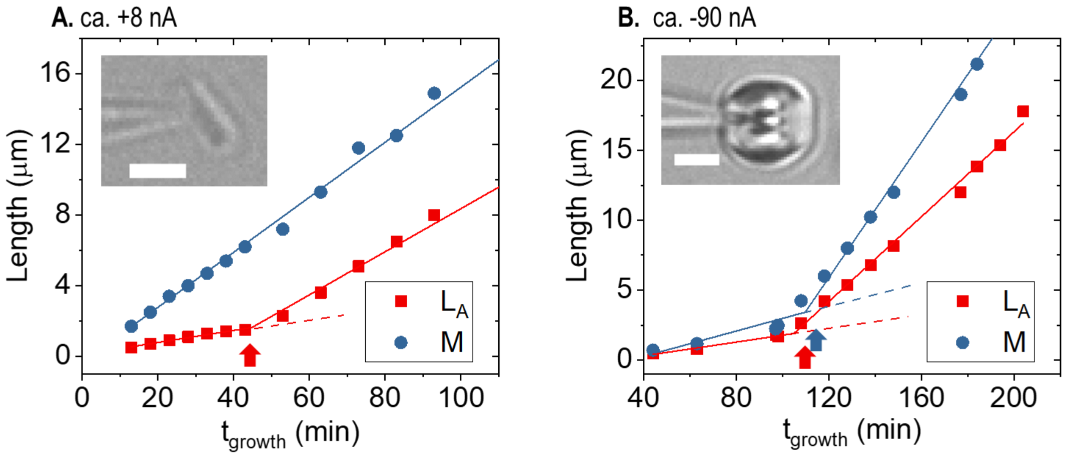

Figure S8. Crystal growth controlled at single-entity levels under different current amplitudes. After nucleation initiated under +0.2 V, the potential was adjusted manually to about +1.0 V (A, ca. + 8 nA), and -0.3 V (B, ca. -90 nA) respectively. Data points were fitted at $R > 0.99$ by piecewise linear regression (breakpoint/s indicated by arrow).

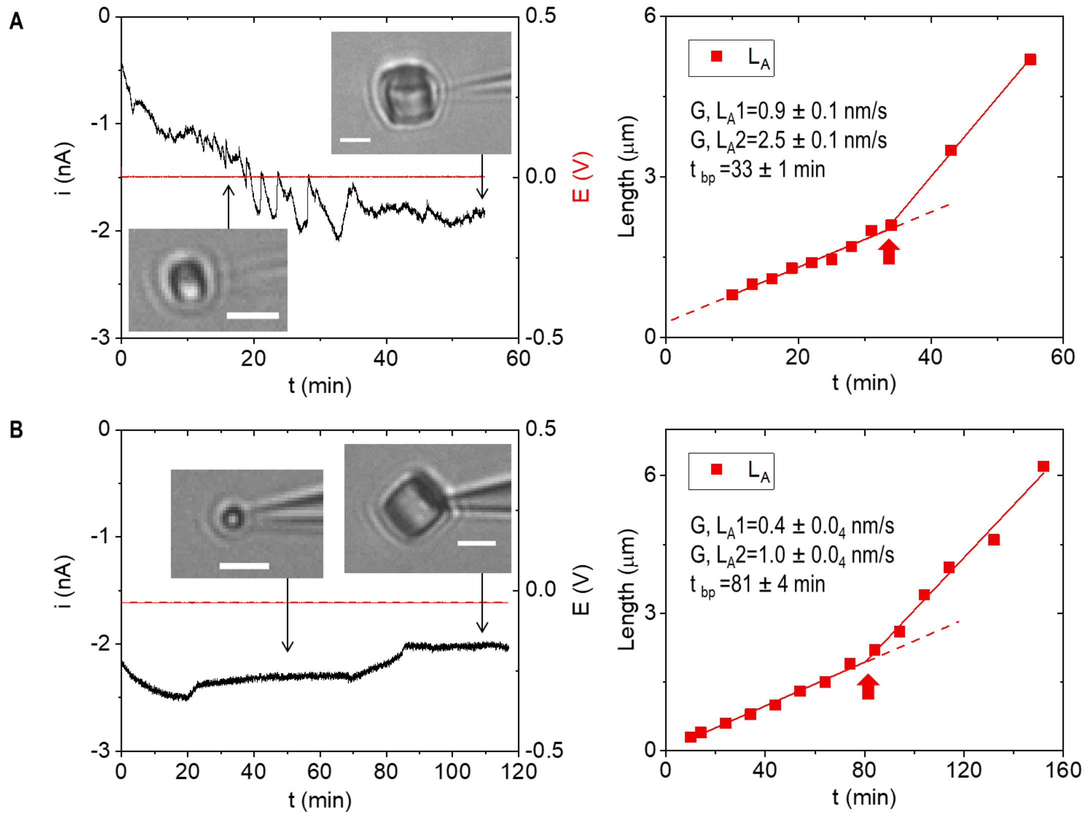

Figure S9. Crystal growth and corresponding current/potential-time curves under less electric filed manipulation. (**A**) 40-nm-radius nanopipette; (**B**) 150-nm-radius nanopipette. After nucleation initiated under +1.0 V (A) or + 0.2 V (B), the potential was adjusted manually to about 0 V. The recorded (streaming) current is mainly from the diffusional flux under the concentration gradient/s across the nanotip. Data points were fitted by linear or piecewise linear regression with R > 0.99. Dashed lines are extrapolated from the linear regression before breakpoint t_{bp} (indicated by arrow). The lengths can only be measured after reaching two pixels or more (single pixel 250 nm; optical limit). Scale bars are 4 μm. Crystal 5 in Table S2 and S5 corresponds to the crystal in B.
